# Contemporary Trends in Frontal Sinus Balloon Sinuplasty: A Pilot Study

**DOI:** 10.51894/001c.123407

**Published:** 2024-09-09

**Authors:** Daniel H. Lofgren, Brandon B. Knight, Carl B. Shermetaro

**Affiliations:** 1 Graduate Medical Education, Otolaryngology McLaren Oakland Hospital, Pontiac, MI, USA; 2 Graduate Medical Education, Otolaryngology McLaren Oakland Hospital, Pontiac, MI, USA.

**Keywords:** Balloon Sinuplasty, Chronic Rhinosinusitis, Frontal Sinus, Complications, Utilization

## Abstract

**Introduction:**

Balloon sinuplasty (BSP) is a common treatment modality used in the management of chronic rhinosinusitis (CRS). Although it has gained popularity, minimal self-reported data on its utilization and complications have been reported. The goal of this study was to describe current practices and complications experienced during frontal sinus BSP.

**Methods:**

An anonymous 20-question online survey was distributed to members of the American Osteopathic Colleges of Ophthalmology and Otolaryngology-Head and Neck Surgery from August 1, 2022, to August 30, 2022. The questions were listed as multiple choice or percentage sliding bars, and data were collected using a commercial online survey service site. Results were reported as frequencies, means, and percentages.

**Results:**

Forty-two respondents participated in the survey, with the majority practicing in the following hospital settings: community (34, 80.95%), hybrid (5, 11.90%), and academic (3, 7.14%). The southeast had the largest proportion of respondents (13, 30.95%), versus the midwest (12, 28.57%), southwest (10, 23.81%), northeast (5, 11.90%), and northwest (2, 4.76%). On average, 50.52% of cases were performed in the hospital setting, 48.50% in-office, and 42.40% in surgery centers. Respondents who primarily used BSP, reported a yearly average of 35.72 cases, a median of 12 cases, and a range of 0-361 cases. Respondents who used BSP with functional endoscopic sinus surgery (FESS), reported a yearly average of 48.62 cases, a median of 31 cases, and a range of 0-189 cases. Nasal packing was utilized both intraoperatively (11.72%) and postoperatively (3.62%). Early complications included postoperative headaches (9.86%), acute bacterial sinusitis (ABRS) (3.52%), and tooth/facial numbness (0.86%). Reported long-term complications included postoperative synechiae (5.10%), orbital complications (0.14%), and skull base complications (0.10%). A previously unreported complication was identified through this study, accidental sphenopalatine fossa dilation.

**Conclusions:**

This study contributes to the growing body of literature on frontal sinus BSP by characterizing utilization and complications from a large otolaryngologic academy.

## INTRODUCTION

Chronic rhinosinusitis (CRS), inflammation of the nasal and paranasal sinus mucosa lasting more than or equal to 12 weeks, reportedly affects over 10% of the population in the United States (US) and Europe; more than 31 million people in the US are affected.^1–5^ Symptoms can include anterior or posterior rhinorrhea, nasal obstruction, hyposmia, anosmia, and facial pain or pressure. Objective findings using nasal endoscopy or computed tomography (CT) are required for the diagnosis.^1–3^ It is further divided into CRS with nasal polyposis (CRSwNP) and CRS without nasal polyposis (CRSsNP), which are treated with a combination of nasal saline, topical or oral steroids, antibiotics, antihistamines, decongestants, immune modulators, aspirin desensitization therapy, and surgical intervention.^3,6–8^ It remains a significant healthcare burden in the US with estimates of 14.4 billion dollars in direct healthcare costs. It also affects approximately 1.7 million school-aged children in the United States, resulting in over $1.8 billion of pediatric healthcare-related expenses per year.^6,9^

Balloon sinuplasty (BSP) has been FDA-approved since 2005 and has been used with increasing frequency over the last 10 years as a treatment option for CRS. It allows for a mucosal-sparing treatment, without destroying the physiologic function of the sinuses by microfracturing and thus dilating the sinus ostia to improve patency and decrease symptomatology, which has been shown to last up to two years.^5,8,10–12^ Currently, there is a limited discussion regarding where BSP fits in the treatment algorithm for patients diagnosed with CRS, and there are only a handful of larger studies looking at postoperative complications and their management.^5,12–14^ In 2014, reported complication and restenosis rates of BSP across all sinuses were noted as 5.26% and 7.89% respectively.^13^ Complications include device failure, epistaxis, hematoma, synechiae, orbital pain, orbit or facial swelling, vision loss, ophthalmoplegia, orbital ptosis, orbital fractures, acute sinusitis, cerebrospinal fluid leak (CSF), pneumocephalus, dural exposure, intracranial bleed, meningitis, and headache among others.^5,12–14^ Hur et al. reported data on 211 BSP-related adverse events from 2008-2018 through an open FDA database and tracked patient (48.3%), device (47.9%), and packing-related (3.8%) adverse events. Of the patient-related adverse events, the most common complications were cerebrospinal fluid (CSF) leak (n = 37, 36.3%), eye swelling (n = 30, 29.4%), and epistaxis (n = 12, 11.8%). The frontal sinus was the most common site of CSF leak (67.6%) and the second most common sinus related to periorbital swelling (20%).^5^

Wright et al. evaluated a separate open database to understand the risks associated with BSP and found 78 adverse events from 2015 to 2018. Of the 78 events, 44 were device-related malfunctions (56.4%), 28 were patient injuries (35.9%), and six were cases of device malfunction and device injury (7.7%). The frontal sinus was the most implicated paranasal sinus (62.7%). In regard to specific complications, skull base injury was seen in 23 cases (30.7%), orbital injury in 12 (15.4%), epistaxis in five (6.4%), sinus infection in one (1.3%), and other complications in three (3.8%).^12^

In regards to the location of BSP treatments, the frontal sinuses have been shown to have the highest complication rates and are associated primarily with CSF leaks, whereas the maxillary sinuses were associated with CSF leaks along with pre-and-post septal injuries, and the sphenoid sinuses were associated with severe epistaxis.^5,12,13^ Interestingly, only one database review reported data on the specific causes of adverse events, showing complicated anatomy in 14.1% of cases and surgeon error in 9.0% of cases.^12^

To the authors’ knowledge, no studies have provided self-reported physician data on the subject, as most data come from insurance claims or open government databases. Nor are there any studies focusing on the frontal sinuses themselves. With the frontal sinuses being one of the most common sites of complications and given the lack of self-reported data in the literature, we aimed to further investigate current trends, utilization, and complications of frontal sinus BSP.

## METHODS

### Study design

This was a cross-sectional observational study. This manuscript was reviewed by the McLaren Health Care Institutional Review Board and met the criteria for exemption under protocol number 2022-0012.

### Study population and study sample

This study population consisted of approximately 660 members of the American Osteopathic Colleges of Ophthalmology and Otolaryngology-Head and Neck Surgery (AOCOO-HNS) members. The study sample included only board-certified active otolaryngologic members of the AOCOO-HNS. Current fellows, residents, and medical students were excluded from the study.

### Measurements

The authors designed a 20-item survey based on the literature and current guidelines and practices. The survey assessed frontal sinus balloon sinuplasty utilization and its complications. Questions were listed as multiple choices or a percentage sliding bar. The survey was reviewed by expert peers for content validity.

### Recruitment of participants

An email was sent to AOCOO-HNS members inviting them to participate in the study. The email included the purpose of the study and a link to the online survey. The survey was voluntary and anonymous. A reminder email was sent two weeks after the initial distribution. Answers to the survey were collected from August 1, 2022, to August 30, 2022. The data were collected using a commercial online survey service site (Qualtrics, Provo, UT).

### Statistical analysis plan

Results are reported as frequencies, means, median, numbers, and percentages. Response alternatives for some questions were not mutually exclusive, and participants could choose more than one choice, and for this reason, the aggregate total could exceed 100%.

## RESULTS

***Characteristics of the study sample***. Forty-two respondents participated in the survey, with 29 respondents fully completing the survey (67.4%). Thirty-three respondents were general otolaryngologists (78.57%) and the remaining nine were fellowship-trained with the following backgrounds: Allergy-Immunology (4.76%), Pediatric (4.76%), Rhinology (4.76%), Otology/Neurotology (4.76%), and Head and Neck Oncology (2.38%). Most respondents practiced in a community setting (80.95%), 11.90% practiced within a hybrid model, and 7.14% in an academic setting. Regarding location, 30.95% of respondents performed balloon sinuplasty in the southeast, compared with the Midwest (28.57%), the Southwest (23.81%), the Northeast (11.90%), and the Northwest (4.76%) (Table 1).

**Table 1.** Distribution of Participants’ Characteristics (N = 42)

**Characteristic**	**Number**	**Percent**
Training Background		
Allergy and Immunology	2	4.76
General Otolaryngology	33	78.57
Head and Neck Oncology and Microvascular Surgery	1	2.38
Pediatric Otolaryngology-Head and Neck Surgery	2	4.76
Otology/Neurotology and Lateral Skull Base Surgery	2	4.76
Rhinology and Anterior Skull Base Surgery	2	4.76
Practice Type		
Academic	3	7.14
Academic/Community Hybrid	5	11.90
Community	34	80.95
Location		
Midwest	12	28.57
Northeast	5	11.90
Northwest	2	4.76
Southeast	13	30.95
Southwest	10	23.81

***Location of BSP Utilization*.** The utilization of BSP varies by surgical location (Table 2). On average, 50.52% were performed in the hospital setting followed by in-office (48.50%) and within a surgery center (42.40%). The average percentage usage of BSP as a supplement to functional endoscopic sinus surgery (FESS) for CRSsNP was 55.27% vs. 52.29% in CRSwNP.

**Table 2.** Distribution of Location of Use of Frontal Sinus Balloon Sinuplasty Location

**Location**	**Percent** **(N = 42)**
Locations BSP Performed - In-office	48.50
BSP Performed - Surgery Center	42.14
BSP Performed - Hospital	50.52
Percent of cases BSP Supplement to FESS in Chronic Rhinosinusitis without nasal polyposis	55.27
In Chronic Rhinosinusitis with nasal polyposis	52.29

BSP = Balloon sinuplasty; FESS = functional endoscopic sinus surgery

***Frequencies of Cases Reported*.** Balloon sinuplasty was reported as a primary procedure for an average of four cases per month (range of 0-31) (Table 3). For primary BSP frontal sinus disease, surgeons reported an average of 36 cases per year (range 0-361). Regarding using BSP in a hybrid procedure (FESS primarily and BSP secondarily), participants reported an average of 5.03 cases per month (range of 0-20), and an average of 48.62 cases per year (range of 0-189) (Table 3).

**Table 3.** Frequencies of Cases Reported by Study Participants (N=29).

	**Mean**	**Median**	**Mode**	**Range**
Number of hybrid (FESS and BSP) frontal sinus cases performed *per month*	5.03	3	2	0 - 20
Number of hybrid (FESS and BSP) frontal sinus cases performed *per year*	48.62	31	100	0 - 189
Primary (BSP Only) frontal sinus balloon sinuplasty procedures *per month*	4.03	1	0	0 - 31
Primary (BSP Only) frontal sinus balloon sinuplasty procedures *per year*	35.72	12	2	0 - 361

BSP = Balloon sinuplasty; FESS = functional endoscopic sinus surgery

***Complications***. The respondents reported complications with the procedure in Table 4. Overall, 15.34% of respondents required utilizing either intraoperative (11.72%) or postoperative (3.62%) absorbable or non-absorbable nasal packing in these BSP cases. They also reported headaches lasting longer than 24 hours in 9.86% of cases, postoperative acute bacterial sinusitis in 3.52% of cases, and tooth/facial numbness in 0.86% of cases. In regards to long-term complications, the group reported postoperative synechiae in 5.10% of patients, orbital complications (orbital wall fracture, ophthalmoplegia, ophthalmology, diplopia, vision loss, globe rupture, preseptal cellulitis, orbital cellulitis, subperiosteal abscess, orbital abscess, cavernous sinus thrombosis) in 0.14% of cases, and skull base complications (pneumocephalus, CSF leak, skull base injury, dural exposure, central nervous system complication, cranial complication) in 0.10% of cases.

**Table 4.** Frequency of Complications Reported by Study Participants (N=29) Complication

**Complication**	**Mean**	**Range**
**Intraoperative Complications**		
Required Intra-Op Packing	11.72	0 - 80
**Postoperative Complications**		
Required Post-Op Packing	3.62	0 - 50
Post-Op Acute Bacterial Sinusitis (within 1 week)	3.52	0 - 25
Post-Op Headache Longer than 24 hours	9.86	0 - 50
Orbital Complications	0.14	0 - 2
Skull Base Complications	0.10	0 - 2
Post-Op Synechia	5.10	0 - 30
Tooth & Facial Numbness	0.86	0 - 15

***Notes**.* Orbital complications included: orbital wall fractures, ophthalmalgia, ophthalmoplegia, diplopia, vision loss, globe rupture, preseptal cellulitis, orbital cellulitis, subperiosteal abscess, orbital abscess, cavernous sinus thrombosis. Skull base complications included CSF leak, pneumocephalus, skull base injury, dural exposure, central nervous system complications, and intracranial complications.

## DISCUSSION

This study presents initial physician-reported data involving the current utilization, operative setting, and complications of frontal sinus balloon sinuplasty from one of the larger otolaryngology academies in the United States. The majority of frontal balloon sinuplasty reported in this study was performed by general otolaryngologists (78.57%) and in community hospital practice settings (80.95%). There was a slight geographic prevalence for performing BSP in the southeast (30.95%), midwest (28.57%), southwest (23.81%), northwest (11.90%), and northeast (4.76%). On average, the physicians surveyed performed more monthly and yearly (5.03 and 48.62) hybrid procedures for the frontal sinus than BSP-only procedures (4.03 and 35.72) respectively. Interestingly, the yearly ranges of performed procedures for both hybrid and BSP-only cases were 0-189 cases and 0-361 cases respectively. Frontal balloon sinuplasty was used slightly more for CRSsNP (55.27 %) compared with CRSwNP (52.29%).

Based on the surgeons’ responses, most BSP-only cases were performed in the hospital setting (50.52%) compared with in-office (48.50%), or in a surgical center (42.14%), likely because we looked primarily at the frontal sinus which is traditionally more difficult to treat and is close in location to the orbit and skull base. In comparison, Chaaban and colleagues [2018] reported revision outcomes, operative setting, and complications for both BSP and FESS, from a large national insurance database. As for the operative setting, 0.74% of FESS cases were performed in the office compared with hybrid (13.37%) and BSP-only (86.53%), although their data includes intervention into all paranasal sinuses. Of note, they reported a revision rate of 7.89% in BSP, 15.15% in hybrid, and 16.85% in FESS, which they attributed to the more severe sinonasal disease in the FESS/Hybrid patients.^13^ Their overall FESS complication rate of 7.35% was reported, compared with BSP (5.26%). In the BSP-only group, the following complications were reported: orbital (2.95%), bleeding (2.03%), and skull base/CNS (0.35%).^13^ Compared to our findings of orbital complications (0.14%), and skull base/CNS complications (0.10%). Our study did report the use of both intra-operative and post-operative nasal packing, which has not been reported in prior literature, but it is difficult to determine if this was due to epistaxis complications or the physicians’ perioperative protocol. We also reported acute postoperative complications including headaches lasting longer than 24 hours (9.86%), postoperative synechiae (5.10%), acute bacterial sinusitis (3.52%), and tooth/facial numbness (0.86%). One respondent did note a complication of “accidental sphenopalatine fossa dilation” which has not been described in the literature.

Sillers and colleagues [2015] performed a claims database analysis of patients undergoing in-office BSP, of all paranasal sinuses, to identify comorbidities, adverse events, and utilization in 2015. They reported similar percentages of both orbital (0.3%) and CSF (0%) complications to our study and included data on the post-operative bleed rate (1.1%), and reoperation rate within 6 months (3.5%).^14^

We do acknowledge some notable limitations to this study including a low number of respondents who fully participated in the survey, polling respondents from only one American academy, physician preferences, heterogeneity of operative and post-operative protocols, and possible recall bias from the respondents. We hope to build on this study by surveying a larger number of respondents within other otolaryngologic academies around the world.

The future goals of this project would be to evaluate and identify frontal BSP revision rates, other complications (device malfunction, failed cannulation, ostial restenosis), image guidance use, and physicians’ reasoning for their chosen operative setting with a large survey population.

## CONCLUSION

Although further investigations are needed, the present study contributes to the growing body of literature on frontal sinus balloon sinuplasty by, for the first time, reporting and characterizing utilization and complications from a large US otolaryngology academy. We hope to build on this study by surveying a larger number of respondents within other otolaryngologic academies around the world.

## References

[ref-355623] Hirsch A. G., Stewart W. F., Sundaresan A. S., Young A. J., Kennedy T. L., Greene J. S.. (2017). Nasal and sinus symptoms and chronic rhinosinusitis in a population-based sample. Allergy.

[ref-355624] Hastan D., Fokkens W. J., Bachert C., Newson R. B., Bislimovska J., Bockelbrink A., Bousquet P. J., Brozek G., Bruno A., Dahlén S. E., Forsberg B., Gunnbjörnsdóttir M., Kasper L., Krämer U., Kowalski M. L., Lange B., Lundbäck B., Salagean E., Todo-Bom A., Tomassen P., Toskala E., van Drunen C. M., Bousquet J., Zuberbier T., Jarvis D., Burney P. (2011). Chronic rhinosinusitis in Europe--an underestimated disease. A GA²LEN study. Allergy.

[ref-355625] Bachert C., Marple B., Schlosser R. J.. (2020). Adult chronic rhinosinusitis. Nature Reviews Disease Primers.

[ref-355626] Rosenfeld R. M., Piccirillo J. F., Chandrasekhar S. S., Brook I., Ashok Kumar K., Kramper M., Orlandi R. R., Palmer J. N., Patel Z. M., Peters A., Walsh S. A., Corrigan M. D. (2015). Clinical practice guideline (update): adult sinusitis. Otolaryngol Head Neck Surg.

[ref-355627] Hur K., Ge M., Kim J., Ference E. H. (2020). Adverse Events Associated with Balloon Sinuplasty: A MAUDE Database Analysis. Otolaryngol Head Neck Surg.

[ref-355628] Bhattacharyya N. (2021). Contemporary Incremental Healthcare Costs for Chronic Rhinosinusitis in the United States. Laryngoscope.

[ref-355629] Sundaresan A. S., Hirsch A. G., Young A. J., Pollak J., Tan B. K., Schleimer R. P., Kern R. C., Kennedy T. L., Greene J. S., Stewart W. F., Bandeen-Roche K., Schwartz B. S. (2018). Longitudinal Evaluation of Chronic Rhinosinusitis Symptoms in a Population-Based Sample. J Allergy Clin Immunol Pract.

[ref-355630] Lofgren D. H., Shermetaro C. (2020). StatPearls.

[ref-355631] Gilani S., Shin J. (2017). The burden and visit prevalence of pediatric chronic rhinosinusitis. Otolaryngol Head Neck Surg.

[ref-355632] Levine H., Rabago D. (2011). Balloon sinuplasty: a minimally invasive option for patients with chronic rhinosinusitis. Postgrad Med.

[ref-355633] Weiss R. L., Church C. A., Kuhn F. A., Levine H. L., Sillers M. J., Vaughan W. C. (2008). Long-term outcome analysis of balloon catheter sinusotomy: two-year follow-up. Otolaryngol Head Neck Surg.

[ref-355634] Wright A. E., Davis E. D., Khan M., Chaaban M. R. (2020). Exploring Balloon Sinuplasty Adverse Events With the Innovative OpenFDA Database. Am J Rhinol Allergy.

[ref-355635] Chaaban M. R., Rana N., Baillargeon J., Baillargeon G., Resto V., Kuo Y. F. (2018). Outcomes and Complications of Balloon and Conventional Functional Endoscopic Sinus Surgery. Am J Rhinol Allergy.

[ref-355636] Sillers M. J., Lay K. F., Holy C. E. (2015). In-office balloon catheter dilation: analysis of 628 patients from an administrative claims database. Laryngoscope.

